# Sex-Specific Screening Mitigates Basal Metabolic Dimorphism Bias and Reveals DIV1 Survival-Associated Metabolic Signatures in *Macrobrachium rosenbergii*

**DOI:** 10.3390/biom16081145

**Published:** 2026-08-06

**Authors:** Yunpeng Fan, Haiqi Zhang, Yang Xu, Xilian Li, Haihua Cheng, Chenhui Wu, Chaofan Yu, Qiang Gao

**Affiliations:** 1 Agriculture Ministry Key Laboratory of Healthy Freshwater Aquaculture, Key Laboratory of Fish Health and Nutrition of Zhejiang Province, Zhejiang Institute of Freshwater Fisheries (Zhejiang Freshwater Fishery Environmental Monitoring Station), Huzhou 313001, China; yunpengfan1994@163.com (Y.F.); zhanghq@zjfish.com.cn (H.Z.); fionxu@foxmail.com (Y.X.); lixilian@126.com (X.L.); m130101022@163.com (H.C.); wch627144866@sina.com (C.W.); 18569853354@163.com (C.Y.); 2 College of Fisheries and Life, Shanghai Ocean University, Shanghai 201306, China

**Keywords:** *Macrobrachium rosenbergii*, serum metabolomics, sex differences, Decapod Iridescent Virus 1

## Abstract

Decapod Iridescent Virus 1 (DIV1) causes severe economic losses in *Macrobrachium rosenbergii* farming. Serum metabolic biomarkers are valuable tools for disease-resistant breeding, yet most existing studies employ mixed-sex samples, and the impact of sexual dimorphism on screening reliability remains poorly quantified. In this study, non-targeted serum metabolomics was performed on 28 healthy *M. rosenbergii* (14 females and 14 males), which were divided into marginally resistant and susceptible groups after DIV1 challenge. We compared results from mixed-sex and sex-specific screening strategies, and separately analyzed the overlap of upregulated and downregulated differential metabolites. Significant basal sexual dimorphism was observed in serum metabolism. Mixed-sex analysis identified 247 differential metabolites, while male-specific and female-specific analyses identified 121 and 161, respectively. Only 26 metabolites showed consistent directional changes across all three comparisons, accounting for 10.5% of total mixed-sex differential metabolites. Nearly 90% of mixed-sex results were either sex-specific signals or independent of sex. Analyses that do not account for sex introduce a large number of sex-unrelated differential markers and mask sex-specific metabolic features, and sex-specific screening is a necessary strategy to obtain reliable metabolic biomarkers for crustacean disease-resistant breeding.

## 1. Introduction

The giant freshwater prawn, *Macrobrachium rosenbergii*, is widely cultured in tropical and subtropical regions of China for its fast growth, large body size and high market value, and has become one of the most economically important freshwater aquaculture crustacean species in the country [1,2]. In recent years, outbreaks of Decapod Iridescent Virus 1 (DIV1) have caused mortality up to 80% in cultured populations, resulting in severe economic losses and becoming a major bottleneck restricting the sustainable development of the industry [3]. At present, there are no effective commercial vaccines or specific drugs for DIV1, and selective breeding for disease resistance is considered the most sustainable and green solution [4,5].

In production practice, *M. rosenbergii* farming includes not only traditional mixed-sex culture, but also all-female and all-male monosex culture modes, which have been gradually promoted in recent years due to their advantages in growth performance and farming management [6,7]. Different culture modes correspond to different breeding targets, and there is an urgent need for targeted disease resistance evaluation tools.

Metabolomics enables comprehensive profiling of small-molecule metabolites in biological samples, which can directly reflect the physiological state of organisms and has been widely used in the screening of aquatic disease resistance biomarkers [8,9]. DIV1 primarily targets hematopoietic tissues and multiple immune organs, and the systemic metabolic and immune disturbances induced by infection can be sensitively captured in circulating hemolymph [10,11]. Accordingly, serum metabolites are highly sensitive to changes in immune status, and can serve as potential indicators for early evaluation of disease resistance [12]. However, most existing studies on crustacean disease resistance metabolomics use mixed-sex samples, and rarely consider the interference effect of sex on metabolic profiles [13]. The applicability of mixed-sex screened biomarkers in monosex culture populations remains unclear.

Sexual dimorphism in immunity and metabolism is a widespread phenomenon in crustaceans [14,15]. The basal metabolic differences between sexes may be superimposed on disease resistance-related differences during mixed-sex analysis, leading to biased results and poor cross-study reproducibility of biomarkers [16,17]. However, the degree of interference caused by sex factors has not been systematically quantified, and the necessity of sex-specific screening for different culture modes remains to be clarified.

In this study, we took DIV1 infection of *M. rosenbergii* as the research model, and systematically compared the results of mixed-sex and sex-specific screening strategies. The purpose of this study was to quantify the interference of mixed-sex on metabolic biomarker screening, reveal the sex specificity of antiviral metabolic features, and provide methodological reference for biomarker development under both mixed-sex and monosex culture modes.

## 2. Materials and Methods

### 2.1. Experimental Animals

Healthy *M. rosenbergii* were obtained from a commercial farm in Huzhou, Zhejiang Province, China. A total of 289 individuals were used in the overall challenge experiment, including 20 unchallenged controls (10 females and 10 males) and 269 DIV1-challenged individuals (164 females and 105 males). All prawns were acclimated in aerated freshwater at 28 ± 1 °C for 7 days before the experiment, and fed with commercial pellet feed twice daily. Only healthy and active individuals with no visible lesions were included. During the 7-day adaptation period, we excluded berried (egg-carrying) females and individuals that had just molted. We also excluded any prawns that molted during the subsequent experimental period to avoid potential metabolic fluctuations caused by molting.

Body length (from the base of the rostrum to the end of the telson) and body weight of each individual were measured before the experiment as baseline phenotypic parameters.

For the metabolomic analysis in this study, 28 individuals with extreme survival phenotypes were selected from the challenged population, with 14 females and 14 males in total. They were assigned to marginally resistant and susceptible groups, with 7 females and 7 males per group, to ensure balanced sex ratio between groups.

### 2.2. Virus Challenge and Experimental Grouping

Decapod Iridescent Virus 1 (DIV1) was provided by the Key Laboratory of Fishery Environment and Aquatic Product Quality and Safety of Zhejiang Province, China, and the viral challenge procedure was performed following the well-established protocols from this laboratory [18]. The 96 h post-infection (hpi) median lethal dose (LD_50_) of the virus was determined to be 3.23 × 10^5^ copies per gram of body weight. Accordingly, the working viral suspension was adjusted to a concentration of 3.23 × 10^8^ copies/mL. The injection dose was calibrated based on individual body weight, with 1 μL of viral suspension administered per gram of body weight via intramuscular injection at the ventral abdominal segment. The control group was injected with an equal volume of phosphate-buffered saline (PBS) at the same injection site.

After challenge, all prawns were individually labeled and reared in separate tanks under the same conditions as acclimation. Mortality was monitored by three trained staff members at approximately 15 min intervals for 7 consecutive days, and the exact time of death was recorded for each individual. All individuals ultimately succumbed to the infection. According to survival time, two extreme phenotypic groups were constructed by selecting the 7 shortest-survival and 7 longest-survival individuals within each sex separately, with 7 females and 7 males per group to eliminate sex ratio bias:

Susceptible group (L): the 7 earliest-dead individuals in each sex, representing the weakest antiviral phenotype.

Marginally resistant group (H): the 7 latest-dead individuals in each sex, representing the relatively strong antiviral phenotype.

A total of 28 individuals were included in the subsequent metabolomic analysis. The body length and weight of each group are presented as mean ± SD, and the survival time is shown as a range. Data were analyzed by multiple *t*-test, and statistical significance was set at *p* < 0.05.

### 2.3. Serum Sample Collection

At 24 h before virus challenge, approximately 200 μL of hemolymph was collected from the ventral sinus of each prawn using a sterile syringe. Immediately after collection, the sample was transferred to a 2.2 cm diameter Petri dish and incubated at 4 °C for 2 h to allow complete clotting. The resulting clot was then cut into small pieces of approximately 0.3 cm using a sterile needle, followed by centrifugation at 2000× *g* for 15 min at 4 °C. The supernatant (serum) was collected and stored at −80 °C until metabolomic analysis.

### 2.4. Non-Targeted Metabolomics Analysis

All LC-MS-based untargeted metabolome detection and raw data preprocessing were conducted by Shanghai Meiji Biomedical Technology Co., Ltd. (Shanghai, China).

Briefly, 50 μL of serum sample was transferred into a 1.5 mL centrifuge tube, and mixed with 200 μL of extraction solution (acetonitrile:methanol = 1:1, *v*/*v*) containing four internal standards (e.g., L-2-chlorophenylalanine at a concentration of 0.02 mg/mL). After vortexing for 30 s, the mixture was subjected to low-temperature ultrasonic extraction for 30 min (5 °C, 40 kHz), then placed at −20 °C for 30 min for sufficient protein precipitation. The sample was centrifuged at 13,000× *g* for 15 min at 4 °C. The supernatant was collected, dried under a gentle nitrogen stream, and reconstituted with 100 μL of reconstitution solution (acetonitrile:water = 1:1, *v*/*v*). After another 5 min of low-temperature ultrasonic extraction (5 °C, 40 kHz), the solution was centrifuged at 13,000× *g* for 10 min at 4 °C. The supernatant was then transferred to sample vials with inserts for subsequent LC-MS analysis.

**Figure A1:**
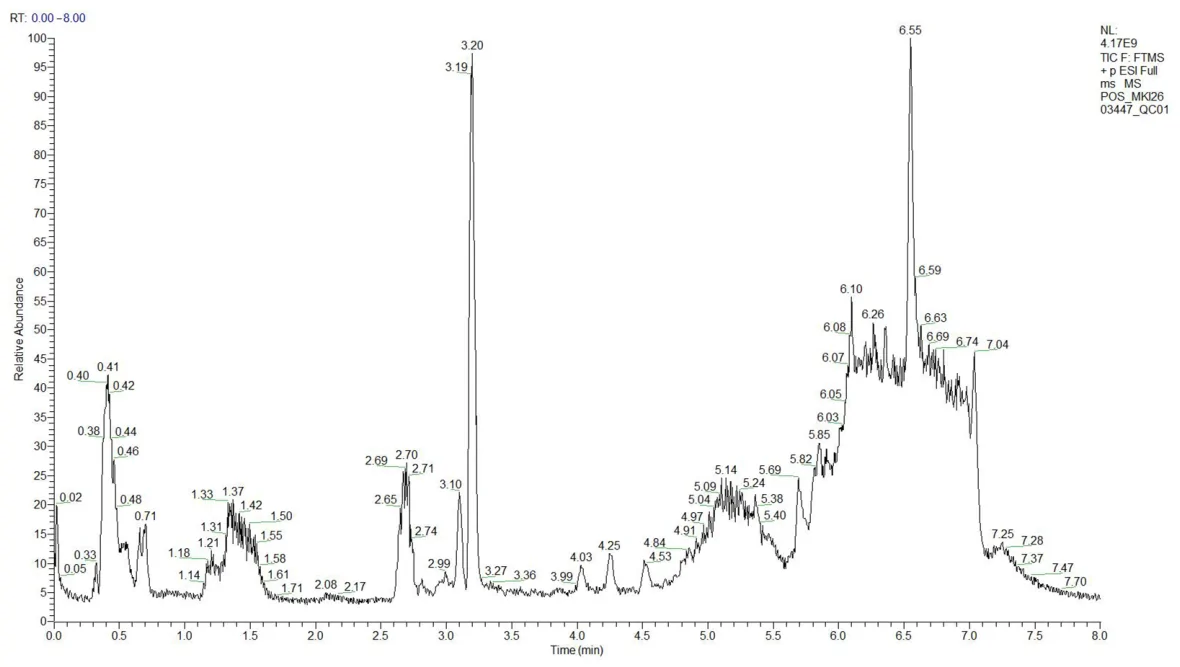
ESI (+) Total Ion Current Plot of Quality Control Samples.

**Figure A2:**
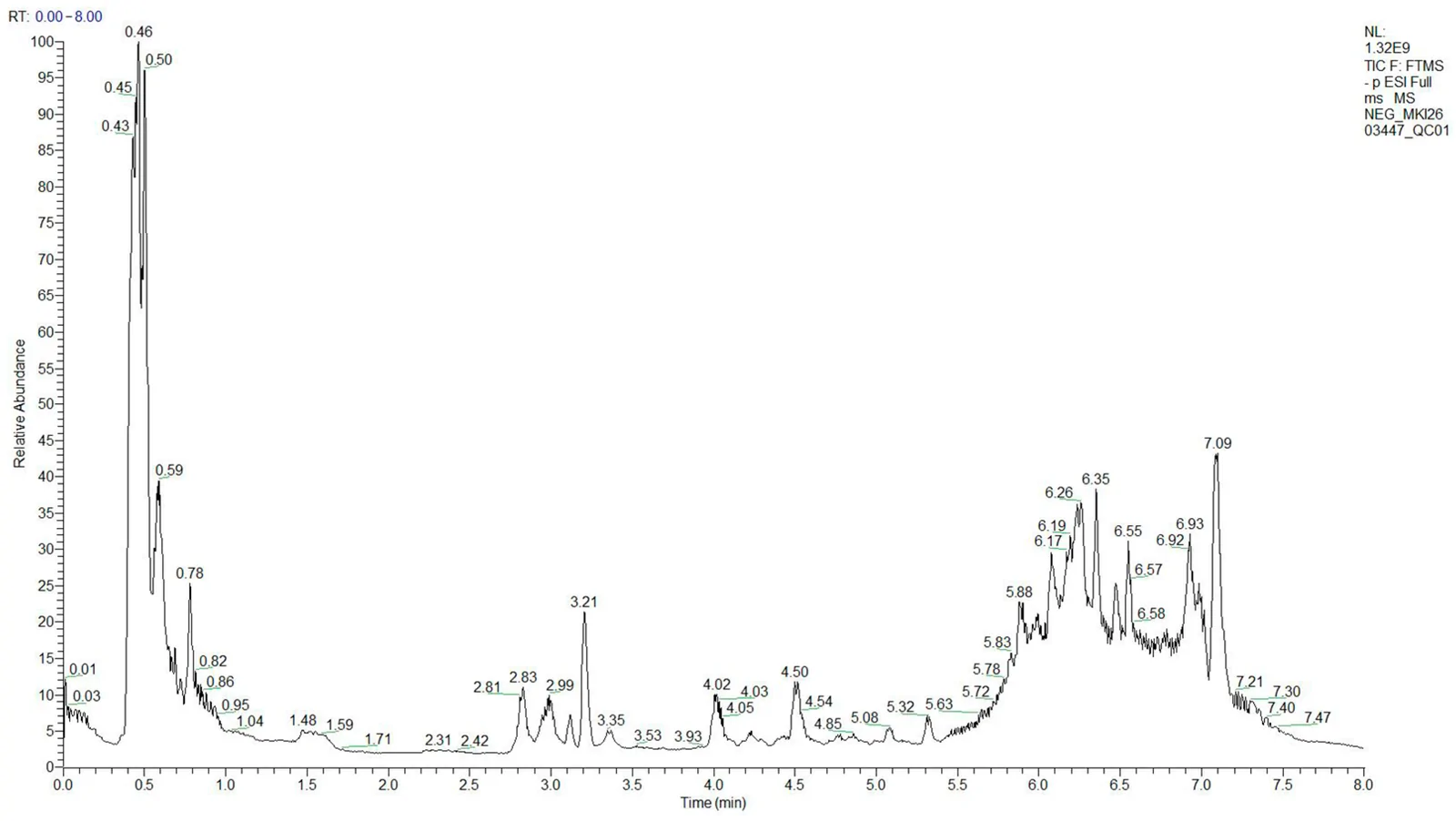
ESI (–) Total Ion Current Plot of Quality Control Samples.

Metabolomic detection was performed using a UHPLC-Orbitrap Exploris 240 system (Thermo Fisher Scientific, Waltham, MA, USA) at Shanghai Meiji Biomedical Technology Co., Ltd. (Shanghai, China). Chromatographic separation was carried out on a Waters HSS T3 column (100 mm × 2.1 mm i.d., 1.8 μm) (Waters, Milford, MA, USA) at a column temperature of 40 °C. The injection volume was 3 μL, and the flow rate was set at 0.40 mL/min. The mobile phase consisted of solution A (0.1% formic acid in water/acetonitrile, 95:5, *v*/*v*) and solution B (0.1% formic acid in acetonitrile/isopropanol/water, 47.5:47.5:5, *v*/*v*/*v*). Instrumental stability was evaluated using total ion current (TIC) overlaps of quality control (QC) samples (Figure A1 and Figure A2).

Mass spectrometry was operated in both positive and negative electrospray ionization (ESI±) modes, with a mass scan range of *m*/*z* 70–1050. The main parameters were set as follows: spray voltage of 3500 V for positive mode and −3000 V for negative mode; sheath gas flow rate of 50 arb; auxiliary heating gas flow rate of 13 arb; ion source heating temperature of 450 °C; stepped collision energy of 20, 40 and 60 V for MS/MS fragmentation.

### 2.5. Data Processing and Statistical Analysis

The data were analyzed through the free online platform the majorbio cloud platform (https://cloud.majorbio.com) (accessed on 3 June 2025) [19].

Raw mass spectrometry data were processed using Progenesis QI v3.0 software (Waters Corporation, Milford, MA, USA). The processing workflow included baseline filtering, peak detection, integration, retention time correction, and peak alignment, yielding a data matrix containing information on retention time, mass-to-charge ratio, and peak intensity.

Metabolite identification was performed via the built-in library search module of the same software. MS and MS/MS spectral information were matched against metabolic databases, with the MS mass tolerance set to less than 10 ppm. Metabolites were ultimately identified according to the matching score of MS/MS spectra. The two main databases used in this study were the Human Metabolome Database (HMDB, http://www.hmdb.ca/) (accessed on 3 June 2025) and METLIN (https://metlin.scripps.edu/) (accessed on 3 June 2025). Metabolite annotation confidence was classified into two levels according to the MS/MS matching source:

Level B(i): High-confidence identification, achieved by precise matching between experimental MS/MS spectra and the in-house validated spectral library, representing the highest identification accuracy in this study.

Level B(ii): Medium-confidence identification, achieved by precise matching between experimental MS/MS spectra and the computationally simulated public theoretical spectral library.

**Figure A3:**
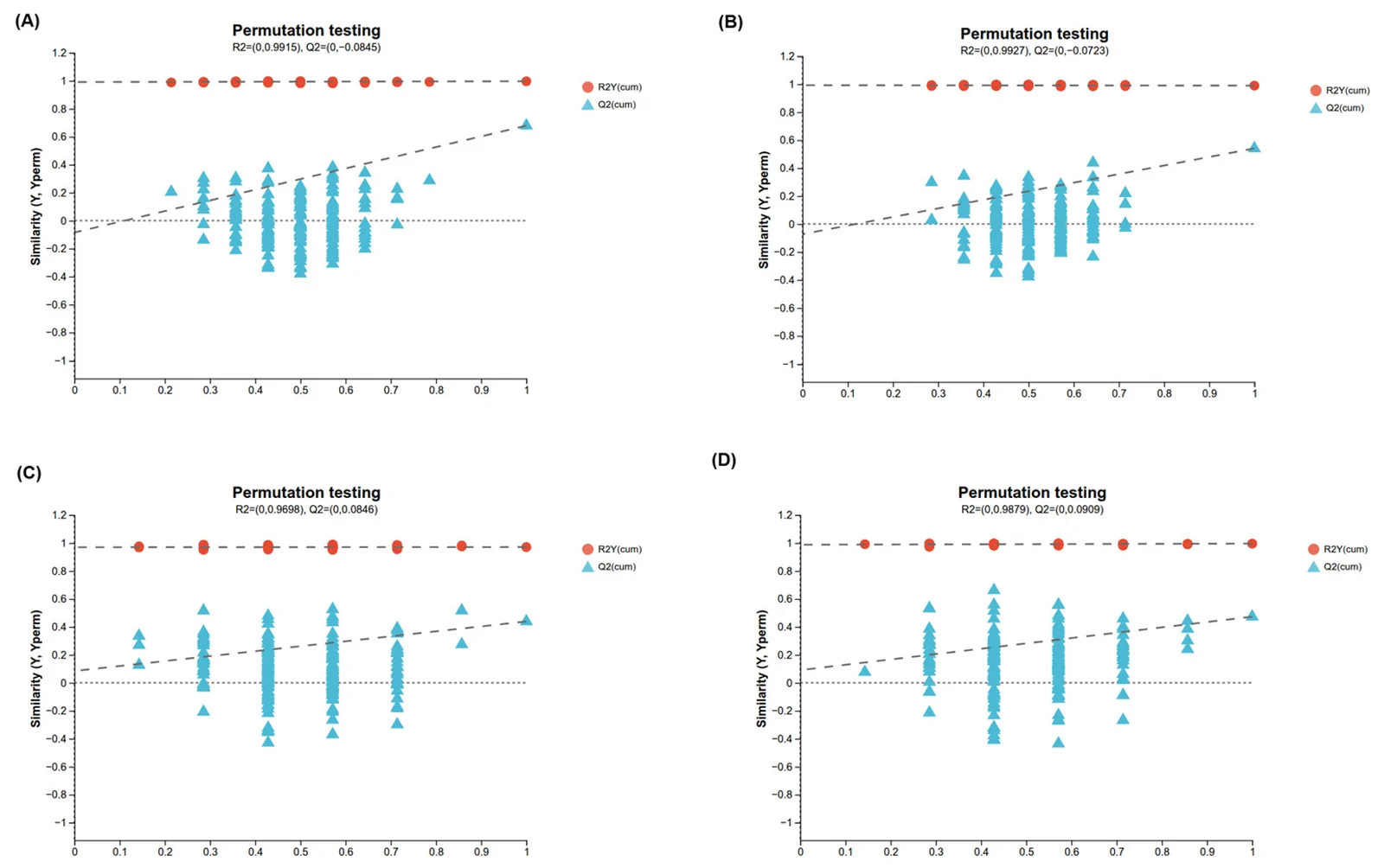
Permutation tests of OPLS-DA models with 200 permutations. (**A**) Female vs. male (basal serum metabolism); (**B**) Resistant vs. susceptible (mixed-sex analysis); (**C**) Resistant vs. susceptible (males only); (**D**) Resistant vs. susceptible (females only). Orange dots represent R^2^Y values, and blue dots represent Q^2^Y values. The two dotted lines indicate the regression lines for R^2^ and Q^2^, respectively.

Orthogonal partial least squares discriminant analysis (OPLS-DA) was conducted using the ropls package (Version 1.6.2) in R to maximize group separation. Model reliability was verified through 200 permutation tests (Figure A3). Significantly differential metabolites were determined based on the variable importance in projection (VIP) values derived from the OPLS-DA model and the *p*-values from Student’s *t*-test. Metabolites with VIP > 1 and *p* < 0.05 were defined as significantly differential metabolites.

Three sets of comparative analyses were performed in sequence:

Basal sexual dimorphism analysis: Comparison between 14 females and 14 males using all 28 samples, without stratification by disease resistance phenotype, to characterize the inherent metabolic differences between sexes.

Mixed-sex screening for disease resistance: Comparison between 14 H and 14 L individuals regardless of sex, representing the conventional analytical strategy.

Sex-specific screening for disease resistance: Separate analyses for males and females, with 7 H vs. 7 L compared within each sex.

The overlap of upregulated and downregulated differential metabolites among the three disease-resistance comparisons was visualized using Venn diagrams. Pathway annotation of differential metabolites was performed against the KEGG Pathway database (https://www.kegg.jp/kegg/pathway.html) (accessed on 3 June 2025) to map the biological pathways involved. Pathway enrichment analysis was carried out using the scipy.stats package (Version 1.0.0) in Python (Version 3.13.2), and Fisher’s exact test was applied to identify the biological pathways most relevant to the experimental treatment.

## 3. Results

### 3.1. Basal Sexual Dimorphism in Serum Metabolism

**Figure 1:**
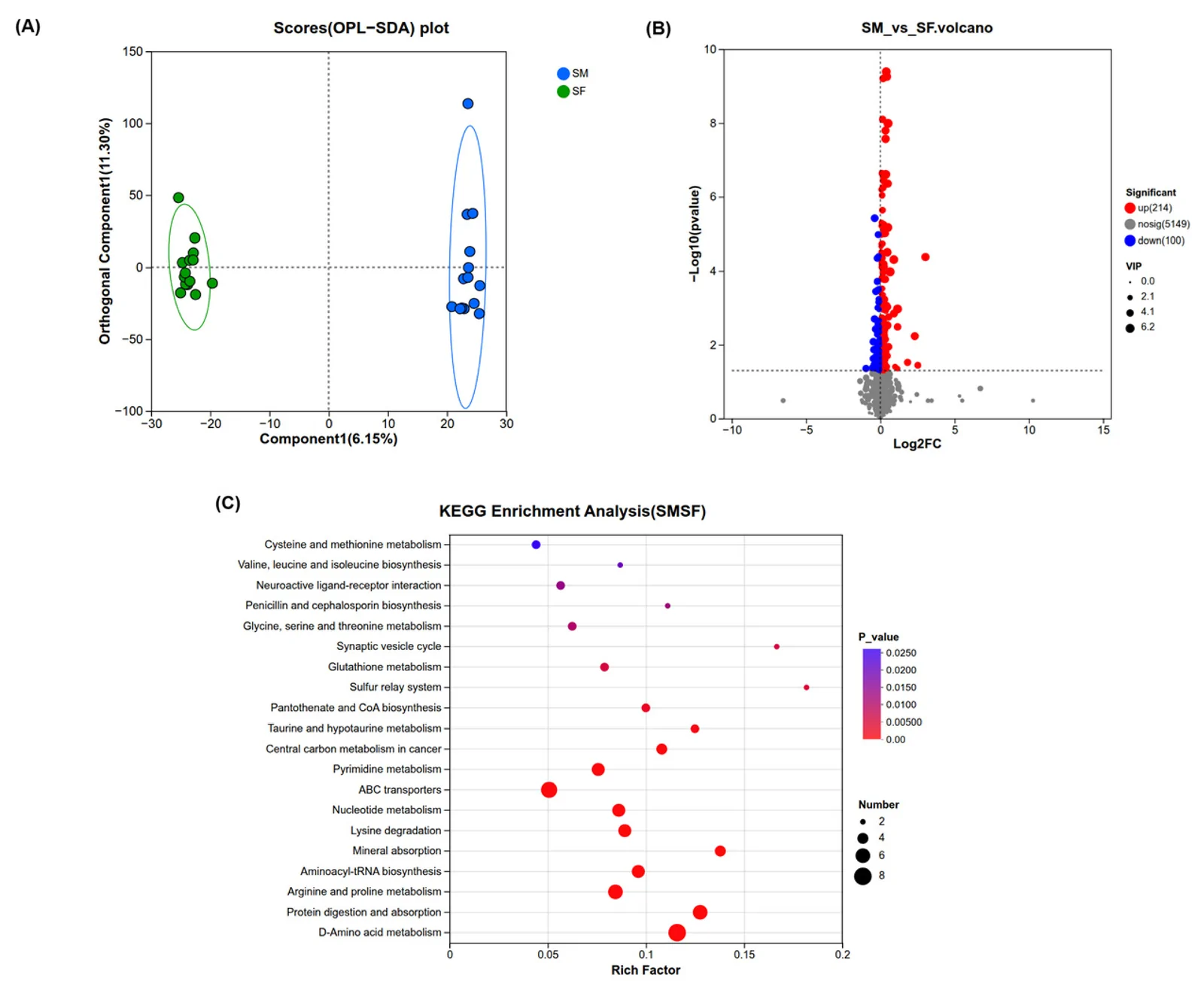
Basal sexual dimorphism in serum metabolism of *M. rosenbergii*. (**A**) OPLS-DA score plot of female (green, *n* = 14) and male (blue, *n* = 14) prawns. (**B**) Volcano plot of basal differential metabolites between females and males. (**C**) KEGG pathway enrichment bubble plot of sex-related differential metabolites.

Based on all 28 serum samples (without stratifying by disease resistance phenotype), OPLS-DA analysis showed a clear separation of serum metabolic profiles between female and male *Macrobrachium rosenbergii* (Figure 1A), with good model fitting and predictive ability (R^2^Y = 0.997, Q^2^ = 0.679).

A total of 314 basal differential metabolites were identified between the two sexes, among which 100 metabolites were significantly more abundant in females and 214 were significantly elevated in males (Figure 1B). KEGG pathway enrichment analysis indicated that these sex-biased metabolites were mainly enriched in amino acid metabolism (e.g., D-amino acid metabolism, arginine and proline metabolism, glycine/serine/threonine metabolism, cysteine and methionine metabolism), nucleotide metabolism (pyrimidine metabolism), ABC transporters, protein digestion and absorption, and glutathione metabolism. Pathways related to amino acid metabolism and membrane transport showed the highest statistical significance, reflecting widespread intrinsic differences in nutrient metabolism and substance transport between sexes (Figure 1C).

These results confirm that prominent basal sexual dimorphism exists in the serum metabolome of *M. rosenbergii*, and sex is an important inherent factor shaping serum metabolic profiles, which may play a large interfering role in the screening of biomarkers for disease resistance.

### 3.2. Survival Phenotype and Experimental Grouping

The 28 individuals selected for metabolomic analysis all died within the observation period, with survival time ranging from 75.02 h to 165.90 h. Basic phenotypic information is shown in Table 1. Males had significantly larger body length and body weight than females (*p* < 0.001), consistent with the sexual dimorphism of *M. rosenbergii*. There was no significant difference in survival time between the male and female members of the susceptible group (*p* = 0.38), and the same was true for the marginally resistant group (*p* = 0.26).

In both males and females, the survival time of the marginally resistant group was significantly longer than that of the susceptible group (*p* < 0.001). The survival time of the susceptible group ranged from 75.02 to 97.93 h, and that of the marginally resistant group ranged from 156.00 to 165.90 h. The clear separation of survival time between the four groups provided a reliable phenotypic basis for subsequent differential metabolite screening.

### 3.3. Disease Resistance-Related Metabolic Features Under Mixed-Sex Screening Strategy

**Figure 2:**
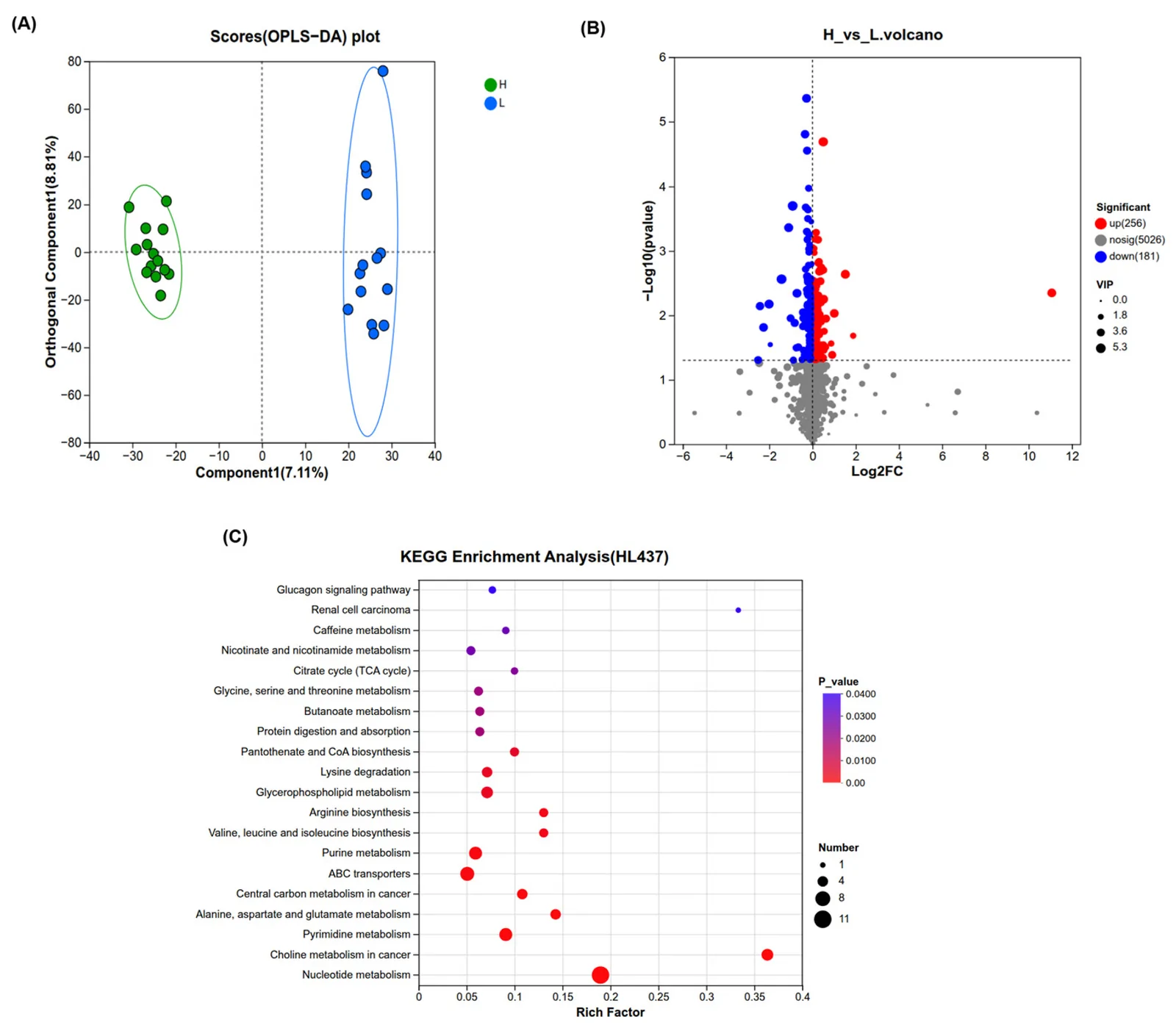
Disease resistance-related metabolic features under mixed-sex screening strategy. (**A**) OPLS-DA score plot of H (green, *n* = 14) and L (blue, *n* = 14) groups with mixed sexes. (**B**) Volcano plot of differential metabolites between H and L in mixed-sex analysis. (**C**) KEGG pathway enrichment bubble plot of mixed-sex differential metabolites.

Under the traditional mixed-sex screening strategy (14 H vs. 14 L), OPLS-DA revealed a distinct separation of serum metabolic profiles between the two groups (Figure 2A), with good model fitting performance (R^2^Y = 0.99, Q^2^ = 0.541).

**Figure 3:**
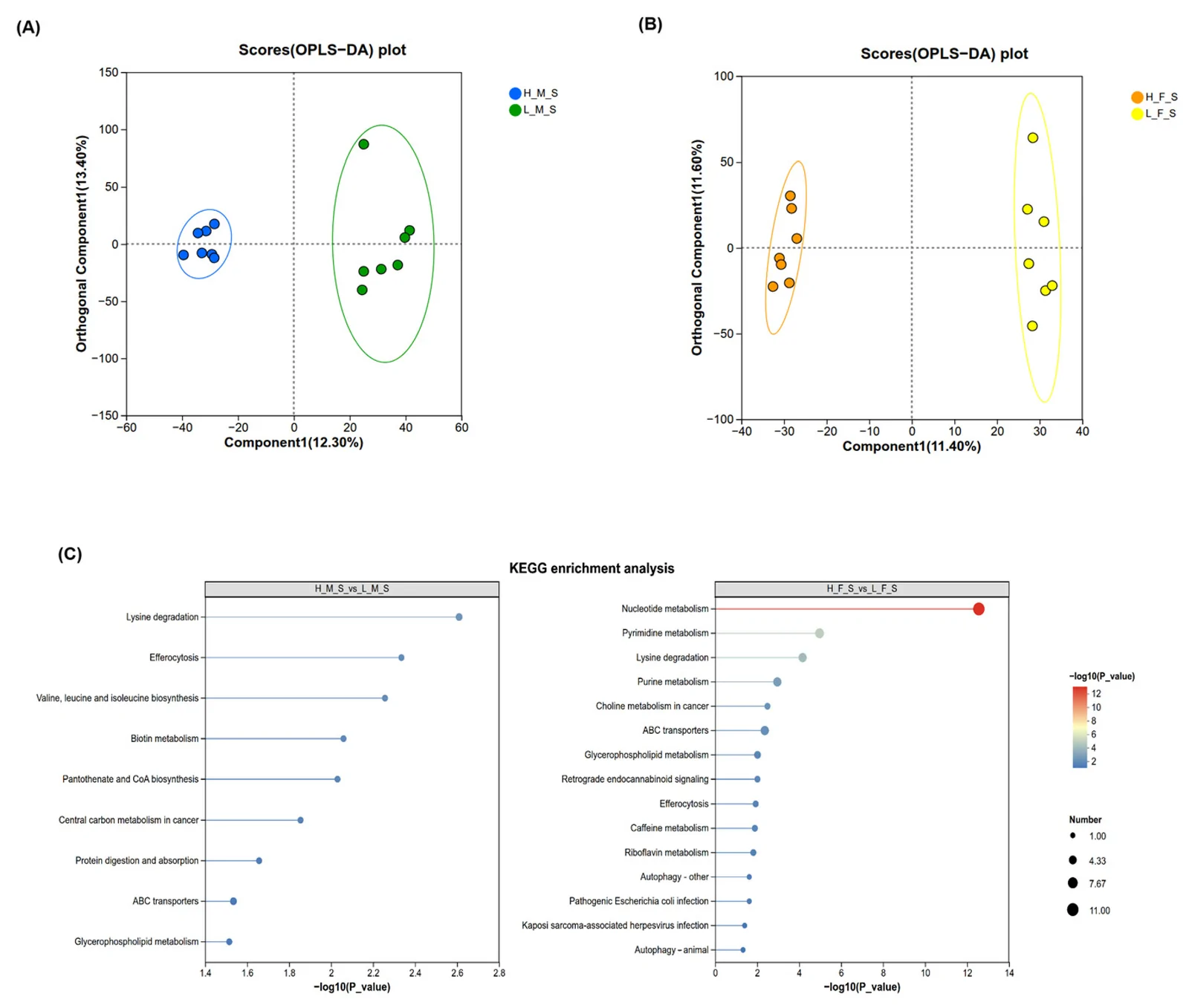
Disease resistance-related metabolic features under sex-specific screening strategy. (**A**) OPLS-DA score plot of 7 H_M_S vs. 7 L_M_S in the male cohort. (**B**) OPLS-DA score plot of 7 H_F_S vs. 7 L_F_S in the female cohort. (**C**) Functional comparison of disease resistance-related enriched pathways between males and females.

A total of 437 differential metabolites were identified, among which 256 were significantly up regulated and 181 were down regulated in the H group compared with the L group (Figure 2B). There are a total of 247 endogenous substances, including 142 that are up regulated and 105 that are down regulated (Figure 3). KEGG pathway enrichment analysis indicated that these differential metabolites were mainly enriched in nucleotide metabolism (including purine metabolism and pyrimidine metabolism), amino acid metabolism (such as alanine/aspartate/glutamate metabolism, arginine biosynthesis, valine/leucine/isoleucine biosynthesis, and lysine degradation), carbohydrate metabolism (including citrate cycle and glyoxylate/dicarboxylate metabolism), glycerophospholipid metabolism, ABC transporters, and pantothenate and CoA biosynthesis. Nucleotide and amino acid metabolism pathways showed the highest enrichment significance, reflecting widespread alterations in energy supply, substance anabolism and membrane transport associated with disease resistance under mixed-sex analysis (Figure 2C).

### 3.4. Disease Resistance-Related Metabolic Features and Pathway Divergence Under Sex-Specific Screening Strategy

When analyzed separately by sex, distinct metabolic signatures associated with disease resistance were observed in both male and female *M. rosenbergii*. In the male cohort (7 H_M_S vs. 7 L_M_S; abbreviations: H = marginally resistant, L = susceptibility, M = male, S = sex-specific grouping), OPLS-DA analysis showed clear separation of metabolic profiles between the two groups (Figure 3A), with good fitting performance (R^2^Y = 0.91) and near the acceptability threshold (Q^2^ = 0.439). A total of 121 differential metabolites were identified, among which 65 were upregulated and 56 were downregulated in the H_M_S group relative to the L_M_S group. In the female cohort (7 H_F_S vs. 7 L_F_S; F = female), the OPLS-DA model (Figure 3B) also exhibited reliable discrimination (R^2^Y = 0.996, Q^2^ = 0.473), with 161 differential metabolites detected; 79 metabolites were significantly elevated and 82 were decreased in the H_F_S group. The slightly lower Q^2^ values in sex-specific analyses were mainly attributed to the reduced sample size per group, and all models were validated by 200 permutation tests to exclude overfitting.

Pathway enrichment analysis further revealed distinct functional characteristics of baseline differential metabolites between the susceptible group and the marginally resistant group of the two genders (Figure 3C). In males, disease resistance-related differential metabolites were mainly enriched in amino acid metabolism (valine, leucine and isoleucine biosynthesis, lysine degradation), metabolism of cofactors and vitamins (pantothenate and CoA biosynthesis, biotin metabolism), glycerophospholipid metabolism, ABC transporters, and protein digestion and absorption. These pathways are tightly associated with energy substrate supply, cellular biosynthesis and antioxidant defense. In females, the differential metabolites were predominantly enriched in nucleotide metabolism (purine metabolism, pyrimidine metabolism), glycerophospholipid metabolism, lysine degradation, ABC transporters, and riboflavin metabolism, which are primarily involved in nucleic acid synthesis, lipid homeostasis maintenance and transmembrane substance transport.

These findings indicate that male and female *M. rosenbergii* harbor distinct baseline metabolic signatures associated with DIV1 survival outcomes: males show pre-infection metabolic profiles enriched in energy metabolism and cofactor synthesis pathways that may support physiological stability upon viral challenge, whereas females exhibit baseline metabolic features dominated by nucleotide metabolism and lipid membrane remodeling pathways linked to better post-infection survival. This widespread sex-specific metabolic divergence further supports the necessity of sex-specific biomarker screening for different culture modes.

### 3.5. Comparative Analysis of Differential Metabolites from Two Screening Strategies

To avoid misclassifying metabolites with opposite change directions as common differential markers, we separately analyzed the overlap of upregulated and downregulated differential metabolites among the three comparisons (mixed-sex, male-specific, female-specific).

#### 3.5.1. Overlap of Upregulated Differential Metabolites

Among the 142 upregulated metabolites identified in mixed-sex analysis, only 4 were consistently upregulated in both male-specific and female-specific analyses, accounting for only 2.8% of the total upregulated metabolites in mixed-sex screening. From the sex-specific perspective, the 4 common upregulated metabolites accounted for only 6.2% of the total upregulated metabolites in males and 5.1% in females.

Notably, all common upregulated metabolites shared by males and females were detected in the mixed-sex analysis; there were no common upregulated markers that could only be detected by sex-specific screening but missed by mixed screening.

**Figure 4:**
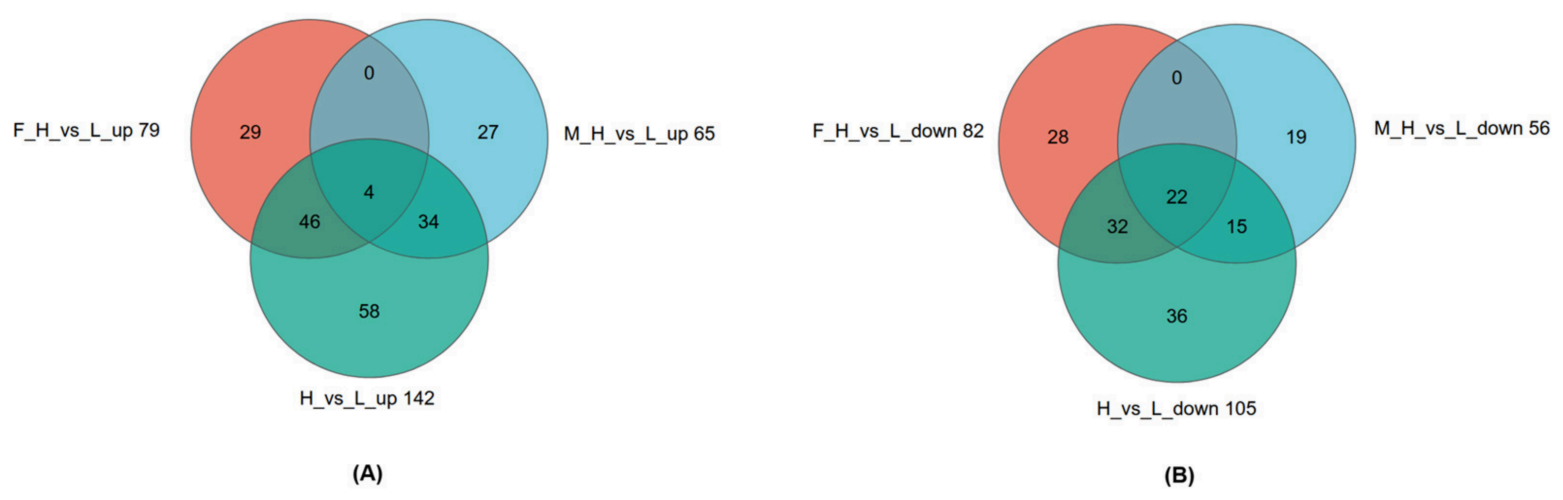
Overlap of upregulated and downregulated differential metabolites among three comparisons. (**A**) Venn diagram of upregulated differential metabolites (higher in H group) from mixed-sex, male-specific and female-specific analyses. (**B**) Venn diagram of downregulated differential metabolites (lower in H group) from the three comparisons.

In addition, 58 metabolites showed significant differences only in the mixed-sex comparison but not in either sex-specific analysis. These signals are mainly derived from the superposition of baseline sexual dimorphism and group differences, rather than real universal resistance-related metabolic changes. 27 metabolites were exclusively upregulated in males, and 29 were exclusively upregulated in females, showing strong sex specificity (Figure 4A).

#### 3.5.2. Overlap of Downregulated Differential Metabolites

Among the 105 downregulated metabolites identified in mixed-sex analysis, 22 were consistently downregulated in both male and female analyses, accounting for 20.9% of the total downregulated metabolites in mixed-sex screening. From the sex-specific perspective, the 22 common downregulated metabolites accounted for 39.3% of the total downregulated metabolites in males and 26.8% in females.

Consistent with the upregulated set, all common downregulated metabolites shared by males and females were covered by the mixed-sex analysis. Additionally, 36 metabolites were significantly downregulated only in the mixed-sex analysis and were not statistically different in the analysis discriminating between the sexes. 19 metabolites were exclusively downregulated in males, and 28 were exclusively downregulated in females (Figure 4B).

#### 3.5.3. Summary of the Two Strategies

Taken together, only 26 metabolites (4 upregulated + 22 downregulated) showed consistent differential expression in the same direction across all three comparisons, accounting for 10.5% of the total differential metabolites in mixed-sex analysis (Figure 4, Table A1 and Table A2). Nearly 90% of the differential metabolites identified by mixed-sex screening were either sex-specific signals or independent of sex.

The vast majority of metabolites associated with DIV1 survival outcomes exhibited strict sex specificity in baseline serum, indicating highly divergent baseline metabolic patterns linked to post-challenge survival between male and female *M. rosenbergii*.

## 4. Discussion

In this study, we systematically compared the performance of mixed-sex and sex-specific screening strategies for identifying baseline metabolic biomarkers associated with DIV1 survival in *M. rosenbergii*, using extreme-phenotype samples selected from a large challenge cohort. Consistent with previous observations in crustacean omics studies [20,21], our results demonstrate that basal sexual dimorphism is a major source of systematic bias in metabolomics-based biomarker research. Mixed-sex screening not only produces a large number of sex-dependent differential signals that lack cross-sex universality, but also masks the highly sex-specific baseline metabolic features linked to post-challenge survival, leading to skewed and poorly reproducible conclusions [16,17].

The most prominent finding is that the conventional mixed-sex strategy yields a high proportion of non-universal differential signals across sexes. Of the 247 differential metabolites identified in the mixed-sex comparison, only 26 (accounting for 10.5%) were consistently detected in both male and female sex-specific analyses, meaning over 90% of the candidates from mixed screening were not genuine universal resistance markers. This pattern is biologically explicable. To the best of our knowledge, this is the first study to systematically characterize profound basal sexual dimorphism in the serum metabolome of *M. rosenbergii*, identifying 314 differentially abundant metabolites mainly involved in amino acid metabolism, nucleotide metabolism and membrane transport. When samples of both sexes are pooled for comparison, these inherent metabolic differences between males and females are superimposed on disease resistance-related variations. Notably, even the 26 shared resistance-associated metabolites identified in both sexes also showed widespread basal sex dimorphism (Table A1 and Table A2). Among the 22 metabolites downregulated in resistant phenotypes, 14 were significantly more abundant in males than females under healthy conditions. As a result, a large proportion of sex-associated metabolites are misidentified as resistance biomarkers, which may be a core reason for the generally poor cross-study reproducibility of metabolic biomarkers in crustacean disease research [9,17].

Further analysis revealed both conserved and sex-specific features of baseline metabolism associated with DIV1 survival between males and females. On one hand, several core pathways were shared between the sexes as baseline metabolic correlates of post-challenge survival, including glycerophospholipid metabolism, ABC transporters and lysine degradation. These pathways are widely documented as conserved metabolic modules of crustacean antiviral immunity, and their pre-infection baseline levels may contribute to differential survival outcomes by supporting core physiological processes such as cell membrane remodeling, transmembrane substance transport and amino acid catabolism during viral infection [22,23]. On the other hand, the enriched functional pathways showed clear sex divergence. In males, resistance-related differential metabolites were specifically enriched in branched-chain amino acid biosynthesis, pantothenate and CoA biosynthesis, and biotin metabolism. These pathways are tightly linked to energy substrate supply, tricarboxylic acid cycle cofactor synthesis and cellular antioxidant defense, supporting the view that male crustaceans prioritize maintaining energy homeostasis and antioxidant capacity to resist viral infection [24,25]. In females, the uniquely enriched pathways were dominated by nucleotide metabolism (purine and pyrimidine metabolism) and riboflavin metabolism, which are primarily responsible for nucleic acid synthesis, DNA damage repair and cellular redox homeostasis [26,27]. Notably, even within the shared pathways such as glycerophospholipid metabolism, the specific differential metabolites showed very low overlap between sexes, implying that the regulatory targets of the same biological pathway also differ between males and females.

This sex-specific metabolic divergence can be well explained by the life history trade-off theory between reproduction and immunity of immune function [28]. Male and female crustaceans have evolved distinct energy allocation strategies among somatic growth, gonadal development and immune defense [29]. Males typically invest more resources in somatic growth and rely more on energy metabolism and antioxidant systems to cope with infection, while females allocate more energy to reproductive output and tend to maintain immune homeostasis via lipid remodeling and nucleic acid repair. After sexual maturity, within-sex variation in growth potential is larger in males than in females. This might be the reason for the lower Q^2^ value in the Male-cohort model. Similar dimorphic immune-metabolic strategies have also been documented in other decapod species such as the Chinese mitten crab and Pacific white shrimp [14,30]. Therefore, a single set of universal markers cannot accurately reflect the disease resistance status of both sexes simultaneously.

From a methodological perspective, our findings strongly suggest that sex must be treated as a key variable in all future crustacean metabolomics studies. The sex-specific screening strategy can effectively eliminate the systematic bias caused by basal sexual dimorphism, and fully capture sex-specific immune metabolic features, which is more compatible with the practical demands of disease-resistant breeding, especially for the increasingly promoted monosex culture modes [7,31]. The 26 common metabolites identified in this study can serve as candidate universal biomarkers, but their stability and universality still require further validation in larger independent cohorts.

This study has several limitations. First, we adopted an extreme phenotype sampling strategy, selecting only the 7 individuals with the longest and shortest survival time within each sex for metabolomic profiling. VIP and *p*-value correction were used instead of FDR. While this design enhances statistical power to detect metabolic differences linked to survival phenotypes, it may inflate the true effect size and restrict the generalizability of findings to prawns exhibiting extreme phenotypes. We openly acknowledge that uncorrected *p*-values may carry a higher false positive risk, and all identified metabolites are preliminary exploratory candidates. Consistent with standard practices in untargeted metabolomics research, our conclusions require further validation in larger, multi-batch independent cohorts [32]. Second, this study is based on non-targeted metabolomics profiling. According to the standard research paradigm of metabolomics, the absolute concentrations and functional mechanisms of the candidate markers remain to be confirmed by targeted quantification and in vivo functional experiments [33]. Third, this study only focused on serum metabolites, and tissue-specific metabolic responses in immune organs such as hepatopancreas and hemocytes can be further explored in future research. Furthermore, body weight and body length are the major confounding variables in this study. Thus, all the sex-associated metabolic differences observed in this study may be mediated by body size or influenced by body size, rather than solely due to gender itself. Nevertheless, our study provides an important methodological reference for the experimental design of crustacean disease resistance biomarker research.

## 5. Conclusions

This study systematically quantified the systematic biasing effect of sex on the screening of DIV1 resistance metabolic biomarkers in *M. rosenbergii.* The results demonstrate that mixed-sex screening introduces nearly 90% non-universal differential signals across sexes, and survival-associated metabolic features show both conserved and sex-specific patterns between males and females, with extremely scarce universal biomarkers. The shared core survival-related metabolic pathways mainly involve glycerophospholipid metabolism, ABC transporters and lysine degradation, while male-specific and female-specific metabolic signatures are dominated by energy/coenzyme anabolism and nucleotide metabolism, respectively. Our follow-up work will focus on the verification and functional validation of these candidate markers. In line with previous methodological consensus on crustacean omics research, sex-specific screening is a necessary strategy to obtain reliable and biologically meaningful results, as it can effectively reduce systematic bias caused by basal sexual dimorphism while retaining sex-specific resistance features. This study provides a solid methodological basis for optimizing the experimental design of crustacean disease resistance biomarker screening.

## Figures and Tables

**Table 1:** Baseline phenotypic information of the 28 prawns used for metabolomic analysis.

Group	*n*	Body Length (cm)	Body Weight (g)	Survival Time (h)
Susceptible male	7	15.04 ± 0.70	92.97 ± 6.35	75.02–97.93
Marginally resistant male	7	14.39 ± 0.69	86.28 ± 9.17	158.00–165.90
Susceptible female	7	13.03 ± 0.48	48.89 ± 5.39	77.12–91.50
Marginally resistant female	7	13.33 ± 0.40	50.61 ± 5.77	156.00–164.50
Total	28	13.95 ± 1.02	69.69 ± 21.64	75.02–165.90

## Data Availability

The data directly relevant to this paper are available in the main text or Appendix A. The raw metabolomics data supporting the findings of this study have been deposited in the OMIX, China National Center for Bioinformation/Beijing Institute of Genomics, Chinese Academy of Sciences (https://ngdc.cncb.ac.cn/omix) (accessed on 28 July 2026), with the accession number OMIX018924. These data are publicly accessible, and are also available from the corresponding author upon reasonable request.

## Abbreviations

The following abbreviations are used in this manuscript:

*M. rosenbergii*	*Macrobrachium rosenbergii*
DIV1	Decapod Iridescent Virus 1
hpi	Hours post-infection
KEGG	Kyoto Encyclopedia of Genes and Genomes
SM	Male prawn serum
SF	Female prawn serum
H	Marginally resistant group
L	Susceptible group
H_M_S	Marginally resistant male under sex-specific screening strategy
H_F_S	Marginally resistant female under sex-specific screening strategy
L_M_S	Susceptible male under sex-specific screening strategy
L_F_S	Susceptible female under sex-specific screening strategy
*m*/*z*	Mass-to-charge ratio
VIP	Variable importance in projection
ESI	Electrospray ionization

## Appendix A

**Table A1:** Detailed information of 26 metabolites.

Metab ID	Metabolite	*m*/*z*	Level
metab_36027	5-Hydroxy-N-formylkynurenine	251.0674	B(ii)
metab_22010	Glu-Gly-Leu	318.166	B(ii)
metab_10799	Glu-Thr-Tyr	410.1573	B(ii)
metab_21453	Pro-Asp-Leu	344.1816	B(i)
metab_512	(4E,14Z)-2-Aminooctadeca-4,14-diene-1,3-diol	280.2634	B(i)
metab_17398	Ala-Ile	201.1247	B(i)
metab_4218	Ala-Leu	201.1247	B(i)
metab_35549	Cys-gamma-Glu	267.1132	B(i)
metab_15251	Cytosine	112.0504	B(i)
metab_19713	Glu-Ala-Lys-Asp	460.2057	B(i)
metab_19630	Gly-Ile	189.1233	B(i)
metab_3083	Gly-Val	173.0933	B(i)
metab_17540	Ile-Ser	217.1196	B(i)
metab_36470	Isovalerylcarnitine	246.1699	B(i)
metab_35853	Leu-Arg	288.203	B(i)
metab_11215	Leu-Gly	187.109	B(i)
metab_568	Leu-Ser	217.1196	B(i)
metab_35429	N6,N6,N6-Trimethyl-L-lysine	189.1595	B(ii)
metab_35812	N-alpha-Acetyl-L-lysine	187.109	B(i)
metab_9074	Nepsilon-Acetyl-L-lysine	187.109	B(i)
metab_14829	PIP (6 keto-PGF1alpha/16:0)	987.4828	B(i)
metab_35456	Pro-Leu	227.1404	B(i)
metab_36591	Pro-Val	213.1247	B(i)
metab_19692	Val-Ala	187.109	B(i)
metab_36899	Val-Gly	173.0934	B(i)
metab_4024	Val-Leu-Tyr	394.231	B(i)

**Table A2:** Detailed information of the 26 shared DIV1 resistance-related metabolites and their basal sexual metabolic dimorphism between male and female *Macrobrachium rosenbergii*.

Metab ID	H/L		H_M_S/L_M_S		H_F_S/L_F_S		Regulate	SM/SF		Regulate
	VIP	Log2FC	VIP	Log2FC	VIP	Log2FC		VIP	Log2FC	
metab_36027	3.1177	0.1675	2.1705	0.1564	2.5226	0.1787	yes-up	0.6109	0.0197	no-up
metab_22010	3.2915	0.2643	2.3912	0.2868	2.4674	0.2403	yes-up	1.3736	0.0941	no-up
metab_10799	3.475	0.2553	2.6861	0.2903	2.4788	0.2198	yes-up	1.0133	0.0419	no-up
metab_21453	4.4677	1.5206	3.2725	1.4038	3.3248	1.7049	yes-up	2.075	0.5912	no-up
metab_512	1.4449	−0.0395	1.4075	−0.0423	1.4127	−0.0368	yes-down	2.3855	0.0629	yes-up
metab_17398	1.6617	−0.0542	1.4512	−0.056	1.6477	−0.0519	yes-down	2.2346	0.0703	yes-up
metab_4218	1.6091	−0.0579	1.4195	−0.0584	1.5341	−0.0572	yes-down	2.1504	0.0735	yes-up
metab_35549	3.1019	−0.2067	1.9163	−0.1924	2.7888	−0.2209	yes-down	0.9569	−0.0521	no-down
metab_15251	1.6685	−0.0569	0.9692	−0.0404	1.7666	−0.0733	yes-down	1.1491	−0.0363	yes-down
metab_19713	4.0379	−0.3431	2.7728	−0.3437	3.3801	−0.343	yes-down	1.0111	−0.067	no-down
metab_19630	2.7775	−0.1772	1.9797	−0.1721	2.3832	−0.1819	yes-down	0.5477	0.0165	no-up
metab_3083	1.4279	−0.0519	1.2306	−0.0496	1.6287	−0.0546	yes-down	2.3959	0.0832	yes-up
metab_17540	2.4302	−0.1754	2.1707	−0.1534	2.4526	−0.2018	yes-down	3.798	0.2633	yes-up
metab_36470	2.1367	−0.0834	1.7642	−0.1021	1.4691	−0.0641	yes-down	1.2025	0.0437	no-up
metab_35853	2.8655	−0.1776	2.2309	−0.2011	2.1975	−0.1551	yes-down	1.4723	−0.0774	no-down
metab_11215	1.4644	−0.045	1.3471	−0.0479	1.5262	−0.0418	yes-down	2.3122	0.0679	yes-up
metab_568	1.5169	−0.0592	1.236	−0.0505	1.5456	−0.0681	yes-down	1.9538	0.0703	yes-up
metab_35429	2.213	−0.0806	1.5225	−0.0782	1.8472	−0.0828	yes-down	0.9349	−0.0305	no-down
metab_35812	1.3513	−0.0443	1.2803	−0.0482	1.2383	−0.04	yes-down	2.0506	0.0635	yes-up
metab_9074	1.4239	−0.0463	1.3035	−0.0493	1.397	−0.0432	yes-down	2.102	0.0654	yes-up
metab_14829	4.3263	−0.2748	2.7672	−0.2064	4.046	−0.3481	yes-down	1.6505	0.0985	no-up
metab_35456	1.8346	−0.0843	1.608	−0.0789	1.7989	−0.0904	yes-down	2.5407	0.1127	yes-up
metab_36591	1.4222	−0.0485	1.2846	−0.0517	1.3864	−0.0449	yes-down	2.0374	0.0671	yes-up
metab_19692	1.3837	−0.0402	1.2444	−0.0423	1.3288	−0.0383	yes-down	2.1696	0.0617	yes-up
metab_36899	1.8992	−0.1014	1.7342	−0.0875	2.1202	−0.1169	yes-down	3.4491	0.1778	yes-up
metab_4024	1.6912	−0.0784	1.255	−0.072	1.5186	−0.0848	yes-down	1.6278	0.0727	yes-up
